# Identification of Proteins Interacting with PCSK9 Using a Protoarray Human Protein Microarray

**DOI:** 10.31531/2581-4745.1000120

**Published:** 2019-09-18

**Authors:** Catherine J. Wooten, Sreevidhya T. Krishnaji, Quantil M. Melendez, Dayami Lopez

**Affiliations:** a Department of Pharmaceutical Sciences, Biomanufacturing Research Institute and Technology Enterprise (BRITE), College of Arts and Sciences, North Carolina Central University, Durham, NC 27707, USA; b INSPIRE Faculty, Department of Chemistry, Indian Institute of Science Education and Research, Madhya Pradesh, Bhopal 462066 India

**Keywords:** Protoarray, Protein-protein interaction, Hypercholesterolemia, PCSK9, Metabolic diseases

## Abstract

Proprotein convertase subtilisin-kexin 9 (PCSK9) appears to be involved in multiple processes. A ProtoArray Human Protein Microarray was used to identify proteins interacting with biotinylated PCSK9. Fifteen novel proteins interacting with PCSK9 were identified using this technique. Only two of these proteins, sterol carrier protein 2 and hepatoma-derived growth factor, related protein 3, have known functions. The identification of proteins that could affect the expression/function of PCSK9 is of great interest due to potential implications in personalized medicine for hypercholesterolemic patients.

## Introduction

Hypercholesterolemia, which is a major issue in the United States due to the number of patients that suffer this disease, is well-known to improve with diet, exercise, and in the majority of the case, with the use of anti-hypercholesterolemic treatments [1]. Unfortunately, some hypercholesterolemic patients cannot achieve their recommended target levels for low density lipoprotein (LDL)-cholesterol usually due to resistance to statin [2,3]. Interestingly, a gene that has been identified as responsible for statin resistance is proprotein convertase subtilisin/kexin 9 (PCSK9) [4]. Another group of patients (10–25%) suffer from statin intolerance limiting the use of these drugs in these patients [2,3]. Other side effects reported for statins are diabetes [5,6], reduced mental performance [7], and cataract development [8]. However, these adverse effects usually happen in a small number of patients. PCSK9 is the third gene identified in causing familial hypercholesterolemia (FH) in an autosomal dominant manner [9,10]. Serum levels of PCSK9 are not only associated positively with LDL levels in FH patients but also appear to contribute to the phenotypic severity of the FH disorder [11,12]. Both gain-of-function (GOF) and loss-of-function (LOF) mutations in the PCSK9 gene have been reported. The GOF mutations of PCSK9 cause hypercholesterolemia and a higher risk of atherosclerotic-related diseases [13,14]. The LOF mutations, on the other hand, result in hypocholesterolemia and protection against cardiovascular diseases [15,16].

The majority of the PCSK9 present in the serum comes from the liver, small intestines, and kidneys [17]. This convertase controls serum LDL levels by controlling the expression of the LDL receptor at the plasma membrane, especially in hepatic cells, by promoting receptor degradation in the lysosome [18,19]. PCSK9 also degrades the very low-density lipoprotein (VLDL) receptor, the apolipoprotein (Apo) E receptors 1 and 2 (ApoER and ApoER2), the cluster of differentiation 36 and 81, beta-secretase 1, and the epithelial (NA+) channel [20–23]. High PCSK9 levels appear to lead to or are the consequence of, the accumulation of Apo B-containing lipoproteins in the circulation, obesity, diabetes, inflammation, atherosclerotic plaque development, thrombosis, hypertension, and apoptosis (reviewed in [24]). Also, PCSK9 is upregulated during cerebral ischemia, myocardial infarction, kidney disease, and hepatic cancer [24]. Having PCSK9 is also beneficial since it is involved in brain development, especially the cerebellum, liver regeneration, and prevention of infections [24]. To fully comprehend how PCSK9 can control multiple pathways, we need to continue finding protein partners of PCSK9. The objective of this study was to perform ProtoArray to identify proteins that interact with biotinylated-PCSK9 in vitro. The identification of proteins that may influence the expression/function of PCSK9 could assist in the development of personalized treatment options for hypercholesterolemic patients.

## Materials and Methods

### Biotinylation of rPCSK9 –

Before performing ProtoArray microarrays, recombinant (r) PCSK9 (carrier-free; R&D Systems; Minneapolis, MN) was biotinylated using the EZ Link Sulfo-NHS-SS Biotinylation kit (Pierce Thermo Scientific; Rockford, IL). First, to exchange buffers, 10 μg of rPCSK9 was added to a Zeba™ spin desalting column that has been pre-equilibrated according to the manufacturer’s protocol. Centrifugation was performed at 1000 × g for 2 minutes, and the resulting flow-through that contained the rPCSK9 protein in PBS was used in the next step. Sulfo-NHS-SS-Biotin was prepared by dissolving in ultrapure water at the concentration of 10 mM. Biotinylation of rPCSK9 was done with an at least 40-fold molar excess of Sulfo-NHS-SS-Biotin for 1 hour at room temperature. Filtering through another pre-equilibrated Zeba™ spin desalting column was performed as described above to remove excess biotin. Alternatively, excess biotin was removed by filtering through a 3 kDa Amicon ultra-2 centrifugal unit (Fisher Scientific; Pittsburg, PA). Protein concentrations were measured using a NanoDrop 2000 (Fisher Scientific). A HABA assay (reagents provided with the Biotinylation kit) was used to measure biotinylation levels. Calculations for the HABA assay were performed according to the protocol provided with the assay.

### Protein Electrophoresis –

Equivalent amounts of biotinylated protein samples were denatured in NuPAGE® LDS Sample buffer (Invitrogen ThermoFisher Scientific; Carlsbad, CA) supplemented with 20 mM DTT at 70 oC for 5 minutes. These proteins were then subjected to electrophoresis on precast NuPAGE™ 4–12% Bis-Tris protein gels (Invitrogen) and NuPAGE® MES SDS Running Buffer (Invitrogen). Electroblotting onto nitrocellulose membranes using NuPAGE® Transfer Buffer (Invitrogen) and staining with 0.1% Ponceau S (in 5% acetic acid; Fisher Scientific) to protein loading and transfer were performed using standard methods [25].

### Western Blotting Analysis –

This technique was performed mostly as previously described [25]. Blocking with 2% BSA-TBS was performed for 30 minutes at room temperature. Incubation with the primary antibody mouse anti-PCSK9 (Cayman Chemicals; Ann Arbor, MI; diluted 1:1000) was performed overnight at 4°C. After incubating with the HRP-labeled anti-mouse secondary antibody (Fisher Scientific; diluted 1:2,000) or avidin-HRP (Fisher Scientific; diluted 1:500) for 1 hour at room temperature, the SuperSignal West Pico Chemiluminescence Substrate (Pierce ThermoFisher) was used for detection. Several exposures ranging from 0.5 s to 30 minutes were made using a Kodak Image Station 4000R Pro Imaging System (Bend, OR) and the Carestream Molecular Imaging Software-Standard Edition-v.5.4.2.18893 (New Haven, CT).

### Probing of the ProtoArray for Protein-Protein Interaction –

The blocking (50 mM HEPES pH 7.5, 200 mM NaCl, 0.08% Triton X-100, 25% glycerol, 20 mM reduced glutathione, 1X synthetic block, 1 mM DTT) and washing (1X PBS, 0.1% Tween 20, 1X synthetic block) buffers were prepared according to the manufacturer’s instructions. First, the ProtoArray (PAH0525011; Invitrogen) was set at 4°C for 15 minutes to equilibrate to the new temperature. The array was then placed with barcode facing up into a well of a pre-chilled chambered incubation tray. Blocking buffer was added followed by incubation for 1 hour at 4°C with shaking. While waiting, the biotinylated rPCSK9 protein was diluted to a final concentration between 5 – 50 μg/mL in a 120 μl volume. After discarding the Blocking buffer and briefly placing the array on a paper towel to remove excess buffer, the array was put back into the tray, and the diluted biotinylated rPCSK9 was added directly onto the array starting at the top. Carefully, a LifterSlip (Invitrogen) was placed onto the slide, letting the probe disperse over the array. The array was then incubated for 90 min at 4°C, trying to keep it flat and facing up with no shaking. Cold wash buffer was added after the incubation followed by removal of the LifterSlip using forceps. Washing was performed for 5 min at 4°C with shaking. The washing buffer was removed; washing was repeated for a total of five times. Streptavidin-Dylight 650 conjugate (Pierce ThermoScientific) was prepared by diluting to 1 μg/mL in 5 mL wash buffer. After the last wash, the diluted Streptavidin-Dylight 650 was added to the tray. Incubation was done at 4°C with shaking for 90 minutes. Washing was repeated as before for a total of five times. After the last wash, the array was removed from the tray with forceps and dipped into a 50-mL conical tube filled with room temperature distilled water. The array was dried by centrifuging at 200 × g for 2 min in a 50-mL conical tube covered in foil to protect from light. The array was then placed facing down on a LiCor Odyssey scanner and scanned with the following settings: 337 μm resolution, high quality, 0.0 mm focus offset, 700 channels. The output image was saved as a 16-bit TIFF file. Data from three independent arrays were analyzed using the ProtoArray Prospector Imager/Analyzer software 5.2.3 (Invitrogen). The GAL files corresponding to the specific arrays were downloaded from ProtoArray Central (Invitrogen.com) and used to define the array grids. About 9375 proteins, in duplicate, were included in each array.

## Results and Discussion

**Figure 1:**
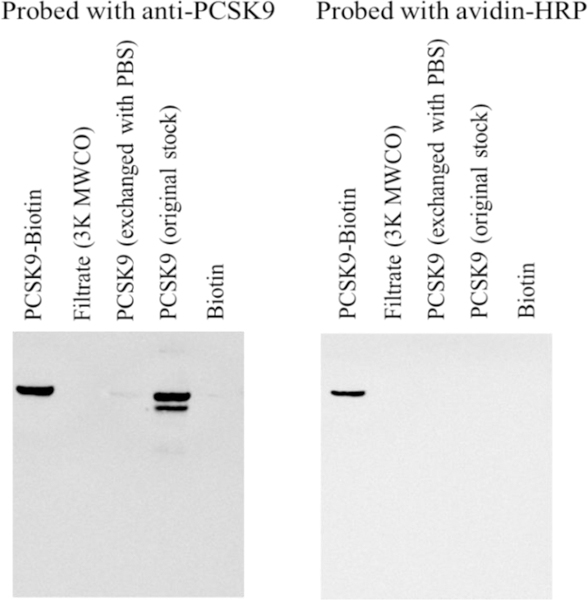
Western blotting analysis of biotinylated PCSK9 before ProtoArray microarray analysis. Recombinant PCSK9 was biotinylated using the EZ Link™ Sulfo-NHS-SS Biotinylation kit as described in Materials and Methods. Electrophoresis followed by Western blotting analysis using either anti-PCSK9 specific antibody or avidin-HRP was done. Typical Western blots are shown. Biotinylated samples were purified using Zeba™ spin columns.

Human rPCSK9 was first biotinylated as indicated in Materials and Methods. Figure 1 shows the results for rPCSK9 biotinylated using 40 molar excess of biotin followed by purification with Zeba™ spin columns. Other samples were loaded onto the gel for comparison. As shown, only the samples containing biotinylated PCSK9 had detectable levels of both PCSK9 (detected using the PCSK9 specific antibody) and biotin (detected using avidin-HRP). Samples containing unbiotinylated PCSK9 (aliquots from PCSK9 exchanged with PBS and the original PCSK9 stock) were only detected with the PCSK9 antibody. Other molar excess concentrations of biotin and alternative methods to remove unreactive biotin molecules were tested as well. Using 200 molar excess of biotin followed by purification with the spin columns (recommended purification method) provided good biotinylation levels that were confirmed as described in Figure 1 and using the HABA assay; each molecule of rPCSK9 was labeled with 15.04 molecules of biotin (data not shown). Using filtration to remove unreactive biotin appeared to result in a rPCSK9 sample with the highest level of biotinylation (226 molecules of biotin/molecule of rPCSK9). However, due to the low detection of PCSK9 in the filtered sample using Western blotting, it was decided to perform the ProtoArray with biotinylated PCSK9 samples purified using the recommended method.

**Figure 2:**
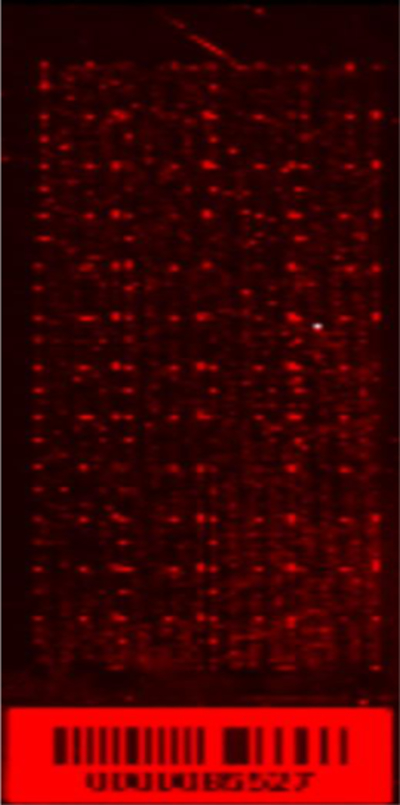
ProtoArray Human Protein Microarray v5.0 probed with biotinylated rPCSK9 protein. Interactions were detected with streptavidin-Dylight 650 dye. Typical whole slide image is shown as acquired using a LiCor Odyssey scanner. This experiment was repeated three times.

Three independent ProtoArray human protein microarrays were probed with biotinylated rPCSK9. Figure 2 illustrates a typical whole slide image as acquired using a LiCor Odyssey scanner. The images were then aligned to grips provided by the GAL files specific for that array (Invitrogen) using ProtoArray Prospector Imager. The data generated were analyzed in the ProtoArray Prospector Analyzer. Table 1 shows the proteins that interacted with biotinylated rPCSK9 in all three ProtoArray microarrays.

None of the proteins in Table 1 have been reported previously as proteins interacting with PCSK9. Furthermore, only the functions of two of these proteins, sterol carrier protein 2 (SCP2) and hepatoma-derived growth factor, related protein 3 (HDGFRP3), have been reported [26,27]. SCP2 is involved in the transport of lipids and cholesterol between different sides of the cellular membrane and is highly expressed in the liver [26,28]. It has been demonstrated that SCP2 levels are significantly reduced in the liver during diabetes, in association with a significant rise in serum cholesterol levels [29]. Thus, it might be possible that PCSK9 works together with SCP2 during diabetes causing deleterious effects that may worsen the disease. The function of HDGFRP3 is less known, but it has been implicated in cell proliferation [29]. The highest expression of HDGFRP3 has been located in testes and brain [27]. Other proteins that interacted with biotinylated PCSK9 in at least one array were adrenomedullin (2), macrophage migration inhibitory factor (2), alcohol dehydrogenase (2), glyceraldehyde-3-phosphate dehydrogenase (2), CYP4A11 (2), caveolin-3 (2), protein phosphatase 2 (1), TNF receptor-associated factor 6 (1), presenilin enhancer 2 homolog (1), thyroid hormone receptor interactor 6 (1), fibronectin-1 (1), glycogen synthase kinase 3 beta (1), platelet-derived growth factor receptor-β polypeptide (1), SERPINF1 (1), and SERPINA3 (1). The number within the parenthesis refers to the number of arrays in which interaction was detected.

Learning more about the different functions of PCSK9 and which proteins can modulate the function of this convertase is critical. Many hypercholesterolemic patients can utilize statins, but those that cannot, will need to rely on PCSK9 inhibitors [30,31]. The main problem with these inhibitors is their cost and the possibility of developing severe side effects [30,31]. The proteins identified herein require further confirmation of their interaction in vivo with PCSK9 and whether they can modify PCSK9’s function. However, they provide a starting point for the identification of novel therapeutic targets to develop personalized treatment options for hypercholesterolemic patients.

## Figures and Tables

**Table 1:** Proteins identified by protein-protein interaction with biotinylated PCSK9 in a ProteinArray Human Protein Array.

Database ID	Mean Signal Used	Mean Z- Factor	Mean Z- Score	Mean CI P- Value	Mean CV	Description
NM_152389.1	6461	−1.94	7.06	0.023	0.64	coiled-coil domain containing 108 (CCDC108), transcript variant 2
NM_201564.1	5212	−0.26	5.28	0.033	0.33	synaptonemal complex central element protein 1 (SYCE1), transcript variant 2
BC059360.1	4557	−1.02	4.41	0.035	0.44	phosphoglucomutase 2-like 1 (PGM2L1)
BC005911.1	4896	−1.89	5.47	0.026	0.89	sterol carrier protein 2 (SCP2)
NM_145177.1	5777	−0.48	6.00	0.043	0.24	dehydrogenase/reductase (SDR family) X-linked (DHRSX)
BC005930.1	5206	−3.04	4.36	0.029	0.79	CD58 molecule (CD58)
BC019102.1	5923	−0.42	6.26	0.024	0.29	dedicator of cytokinesis protein 8
NM_016074.1	4165	−3.50	3.41	0.035	0.94	bolA homolog 1 (E. coli) (BOLA1)
BC021093.1	3762	−0.29	3.89	0.039	0.21	hippocampus abundant transcript-like protein 1
NM_022497.2	6189	0.10	6.49	0.026	0.22	mitochondrial ribosomal protein S25 (MRPS25), nuclear gene encoding mitochondrial protein
NM_020466.3	5388	−0.30	5.69	0.032	0.32	LYR motif-containing protein 2
NM_015959.1	4369	−0.12	4.53	0.032	0.14	thioredoxin domain-containing protein 14
NM_004527.2	4539	−0.72	4.73	0.028	0.36	mesenchyme homeobox 1 (MEOX1), transcript variant 1
BC050328.1	3464	−2.93	3.15	0.030	0.54	neuroblastoma breakpoint family, member 22 (pseudogene) (NBPF22P)
NM_016073.2	3932	−0.20	4.05	0.038	0.23	hepatoma-derived growth factor, related protein 3 (HDGFRP3)

This experiment was performed as described in Material and Methods.

Data from three independent arrays were analyzed using the ProtoArray Prospector Imager/Analyzer software 5.2.3 and the GAL file corresponding to each specific array.
